# Serum lipids and associated factors of dyslipidemia in the adult population in Shenzhen

**DOI:** 10.1186/s12944-015-0073-7

**Published:** 2015-07-14

**Authors:** Wen-Qing Ni, Xiao-Li Liu, Zhi-Peng Zhuo, Xue-Li Yuan, Jin-Ping Song, Hong-Shan Chi, Jian Xu

**Affiliations:** Shenzhen Center for Chronic Disease Control, No.2021, Buxin Rd., Shenzhen, Guangdong 518020 P.R. China

**Keywords:** Dyslipidemia, Risk factors, Serum lipid, Prevalence

## Abstract

**Background:**

Dyslipidemia is one of the most important independent modifiable risk factors for cardiovascular diseases. The objective of this study was to determine the prevalence and risk factors for dyslipidemia in Shenzhen, a special economic zone and large metropolitan area neighboring Hong Kong.

**Methods:**

A cross-sectional survey of 1,995 adults with a mean age of 46.56 years was conducted between February and July 2011 using a multistage stratified cluster random sampling. All the subjects were administered questionnaires regarding socio-demographic characteristics and other possible factors associated with the prevalence of dyslipidemia. Fasting venous blood samples were collected to assess the lipid profile. Weight, height, waist circumference, and blood pressure were measured.

**Results:**

The mean concentrations of total cholesterol (TC), triglycerides (TG), high density lipoprotein cholesterol (HDL-C), and low density lipoprotein cholesterol (LDL-C) were 5.11 ± 1.15 mmol/L, 1.59 ± 1.47 mmol/L, 1.42 ± 0.33 mmol/L, and 3.22 ± 0.84 mmol/L, respectively. High values of TC, TG, low HDL-C, and high LDL-C were obtained in 14.49 %, 16.14 %, 8.82 %, and 12.13 % of the 1,995 participants, respectively. The prevalence of dyslipidemia was 34.64 %, among which 25.04 % of subjects were aware. Presence of dyslipidemia was significantly associated with increasing age, smoking status, hypertension, diabetes, and body mass index.

**Conclusions:**

High prevalence of dyslipidemia with relative low awareness in Shenzhen was found. A comprehensive strategy is required for the prevention, screening, treatment, and control of dyslipidemia in Shenzhen.

## Introduction

Dyslipidemia is defined as the derangements of one or more of the lipoproteins in blood, such as elevated in total cholesterol (TC), low density lipoprotein cholesterol (LDL-C) and/or triglycerides (TG), or low levels of high density lipoprotein cholesterol (HDL-C) alone [1]. The results of other studies suggested that many persons with dyslipidemia were overtly under-diagnosed and it is estimated that prevalent cases of dyslipidemia in the nine major countries will increase at the rate of 1.76 % per year to surpass 500 million in 2022 [2]. The number of people with dyslipidemia is expected to reach 78 million in the major countries by 2022, of which, one sixth of all cases will occurre in China [2]. The prevalence of dyslipidemia is high and increases continuously in many developing countries as a result of the westernization of diet, obesity, aging of population, reduced physical activity, and other adverse lifestyle changes [3]. Dyslipidemia is one of the most important and modifiable risk factors for cardiovascular diseases, which is also a major cause of morbidity and leads to mortality worldwide [4]. The morbidity and mortality of cardiovascular diseases in China are expected to increase both in absolute number and as a proportion of total disease burden, parallel changes in lifestyle and diet in the next 20 years [5].

Several clinical trials have indicated that the treatment of dyslipidemia is effective in both primary and secondary prevention of cardiovascular events [6, 7]. Treatment of dyslipidemia can reduce the risk of heart disease by approximately 30 % over a 5-year period [8]. Nevertheless, surveys in China have showed poor awareness, treatment and control of dyslipidemia [9, 10]. In order to improve dyslipidemia control, it is critical to understand the characteristics of those who have dyslipidemia. Therefore, new initiatives and resources can be targeted and tailored to the needs of high-risk populations. Studies conducted in different regions of China have measured relationships between potential risk factors and dyslipidemia, however, differences in socioeconomic status, demographic composition, lifestyle, dietary, alcohol intake, and physical activity in Shenzhen suggest the necessity of reevaluating these factors in Shenzhen’s current context [11, 12]. Therefore, the primary goal of present study was to investigate the prevalent status of dyslipidemia in a representative sample of the urban population aged more than 20 years that includes 12 streets from 8 districts of the city of Shenzhen, and its association with other risk factors.

## Materials and methods

### Sampling sites

As China’s first Special Economic Zone to spur economic growth after the near collapse of the socialist centrally-planned economy in 1978, Shenzhen has transformed from an agriculture-based Baoan County into a 21^st^ century metropolis housing over millions of people. Unprecedented social and economic development in Shenzhen has led to changes in patterns of diet, physical activity, and other socio-demographic characteristics that are contributing to large increases in the prevalence of cardiovascular disease and other chronic diseases [13]. Dyslipidemia is a well known risk factor for cardiovascular disease. A prevention measure against the epidemic of dyslipidemia will undoubtedly push down the morbidity and mortality of cardiovascular diseases. The associated factors of dyslipidemia in Beijing, Shaoxing, and Delhi has have been evaluated; however, little data are available in Shenzhen [11, 12, 14].

The present study was conducted as a part of the China Health and Nutrition Survey at the Center for Chronic Disease Control of Shenzhen. In brief, the survey used a multistage stratified cluster random sampling method to select a representative sample of permanent residents in 8 different districts in Shenzhen city. The first stage of sampling was stratified by district and population distribution on the basis of Shenzhen population data from 2010, which involved the random selection of 12 streets from those 8 districts. In the second stage, one residential community was randomly selected from each selected street. In the third stage, about 75 households were randomly selected from the residential communities selected in the earlier stage. In the last stage, eligible family member from each designated household was chosen.

### Study population

Volunteers were recruited from selected households from February 2011 to July 2011. The eligibility criteria were set as following: aged 20 years or above, living in Shenzhen for more than 5 years, men or non-pregnant women, confirmed not to present with cancer, severe liver disease, pancreatitis, polycystic ovarian syndrome, systemic lupus erythematosus, and kidney diseases.

From February 2011 to July 2011, 1,995 participants were recruited according to the eligibility criteria. All participants received information and publicity about the health risk that may result from dyslipidemia exposure. All participants have the chances to receive the health check and biochemical determination results if they provided their telephone number or other contact information. The study received ethnicity approval of the Center for Chronic Disease Control of Shenzhen. Written informed consent was obtained from all participants before the study.

### Questionnaire survey

Structured questionnaires were administered to collect information on potential risk factors for elevated blood lipids, as well as possible factors associated with dyslipidemia. Volunteers were interviewed in person 1 to 2 hours after blood collection and each questionnaire took approximately 20 min to complete. Questions covered general information (age, gender, ethnic, place of residence, employment and marital status), socioeconomic status (household per capita income and education level), health behaviors (drinking habit, level of physical activity, and smoking habit), and personal medical history.

The age groups were classified according to results from Shenzhen 2010 census as 20 to 29 years, 30 to 39 years, 40 to 49 years, 50 to 59 years, 60 to 69 years, 69 to 79 years, and ≥80 years. Employment status was classified into one of the followings: homemaker, employed, unemployed, retired, and others. Marital status was categorized as single, married, widowed, and divorced. Educational level was categorized into 5 groups according to the number of years of education (0, 1–6, 7–9, 10–12, and ≥12 years). Subjects were asked to choose 1 of 10 income categories that best represented the household per capita income for the past 12 months: < 5,000 RMB; 5,000 to 9,999 RMB; 10,000 to 14,999 RMB; 15,000 to 19,999 RMB; 20,000 to 24,999 RMB; 25,000 to 29,999 RMB; 30,000 to 34,999 RMB; 35,000 to 39,999 RMB; ≥ 40,000 RMB; and unclear. The questions regarding smoking, which are described in details elsewhere, permit stratification of participants into categories such as ever-smokers (including: current smokers and ex-smokers) and never smokers [15]. Participants were also defined as habitual drinker (drink once per day or more), non-habitual drinker (six times per week to once per month), or non-drinker (almost never) [16]. In the survey, the term “moderate to vigorous intensity physical activity” was defined as physical activity causing at least some sweating and shortness of breath, whereas the term “light physical activity” was defined as causing no sweating or shortness of breath [17]. Furthermore, physical activity was defined as moderate to vigorous intensity physical activity not less than once a week.

### Physical examination

Anthropometric examinations were administered in the morning after fasting overnight, followed by body measurements, which were taken by trained examiners using a standardized protocol. Height, body weight, waist circumference, and blood pressure of all participants were recorded. Height and weight were measured with the participants wearing light dress without shoes using analog scales. Waist circumference (WC) was measured at the midpoint level of midaxillary line between the 12th rib head and the superior anterior iliac spine, with measuring time at the end of normal expiration. Body mass index (BMI) was calculated by dividing body weight (in kilograms) by the square meters of height. Blood pressure was measured using a mercury sphygmomanometer on the right arm of each of the participants in a comfortable sitting position after a 30 min rest period. Three consecutive blood pressure measurements were performed, and the mean of these three measurements was applied in the subsequent analysis.

### Blood sample collection and analyses

Vein blood samples were collected after 10 to 14 hours of fasting. Each glass automatic hemostix was marked with the volunteer’s identification code and placed into ice in a portable refrigerator before transporting to laboratory. In our laboratory, the blood samples were separated by centrifugation at 1000 × g for 15 min at 4 °C, and then supernatant serum was collected. Serum glucose (fasting blood glucose), TC, TG, LDL-C, and HDL-C concentrations were measured by using commercially available kits (Olympus System Reagents, Olympus Diagnostica GmbH, Clare, Ireland) by autoanalyzer (Olympus AU400 System, Olympus, Tokyo, Japan). Serum TG were measured using the glycerol-3 phosphate oxidase-phenol + 4-aminophenazone enzymatic colorimetric method and TC by the cholesterol oxidase method. HDL-C was estimated in a series of reaction steps using an enzymatic cholesterol reagent after precipitation of non–HDL-C with magnesium/dextran, and LDL-C was estimated using a third-generation direct LDL cholesterol assay. The laboratory variation coefficient of TC, TG, HDL-C, and LDL-C was 1.5 %, 4.3 % , 3.0 %, and 3.9 %, respectively (indicating that long term standardization of TC, TG, HDL-C and LDL-C can be obtained with automated enzymatic methods utilizing commercially available reagents). Furthermore, as measures of quality control and assurance, analytical duplicates, and reagent blanks were routinely included in the analytical sequence for detection of precision and signs of contamination, respectively. In all cases, analyses were performed after the correct values had been confirmed by the instrument. All blood samples were analyzed within 4 hours after being collected.

### Definitions of dyslipidemia, obesity, diabetes and hypertension

Dyslipidemias were defined according to Chinese criteria [18]. High TC was defined as a TC value ≥6.22 mmol/L and hypertriglyceridemia (high TG) was defined as a serum TG level ≥2.26 mmol/L. Low HDL-C was defined as a serum HDL-C level <1.04 mmol/L. High LDL-C was defined as a serum LDL-C level ≥4.14 mmol/L. In the present study, high TC, and/or high LDL-C, and/or low HDL-C and/or high TG, and/or having a history of dyslipidemia disease in the past year, and/or currently receiving treatment with lipid-lowering medications was regarded as dyslipidemia. Awareness of dyslipidemia was defined as answering “yes” to the following question: “During the past year, has a healthcare professional told that you had dyslipidemia?”.

Body mass index (BMI) was used as a measure of body size. Participants with BMI <18.5 kg/m^2^ were classified as underweight, those with BMI of 18.5 to 23.9 kg/m^2^ as normal weight, those with BMI of 24 to 27.9 kg/m^2^ as overweight, and those with BMI of ≥28 kg/m^2^ as obese [19]. Two sets of cut offs were used to define central obesity in terms of waist circumference. These were >95 cm for men and >90 cm for women on the basis of specific guidelines for central obesity [19].

Hypertension was defined as mean systolic blood pressure ≥140 mmHg and/or diastolic blood pressure ≥90 mmHg, and/or self-reported antihypertension treatment in the last 2 weeks. Diabetes was defined as fasting blood glucose (FBG) ≥7.0 mmol/L, and/or self-reported treatment of diabetes with antidiabetes medication in the previous 2 weeks.

### Statistical analyses

The mean and standard deviation of continuous variables were expressed as mean ± SD, and percentage was calculated for categorical variables. Age and other anthropometric examination characteristic differences between groups defined by gender were evaluated using Student's *t*-test. The chi-squared test or Fisher’s exact test was used to determine significant differences in proportions among categorical variables. Normal distribution of the serum lipid concentration was tested by Kolmogorov-Smirnov test, and showed a highly skewed. Changes in outcome measures between the different groups not following normal distribution were tested by Mann-Whitney *U* test or Kruskal-Wallis H test for independent variables.

The univariate and multivariate logistic regression models were used to investigate the probability to having dyslipidemia according to socio-demographic and other variables. Statistical analysis of the data was performed by Statistical Package for Social Sciences (SPSS) version 21.0 software (SPSS Inc., Chicago, IL, USA). A *P* value of <0.05 was considered to be statistically significant. We estimated age standardized prevalence rate, and the standard population used in the direct method was the Shenzhen 2010 census population.

## Results

### Socio-demographic and other characteristics of participants

The Socio-demographic and other characteristics of the participants are summarized in Table 1. Of the 1,995 participants, 44.36 % (*n* = 885) were men, 55.64 % (*n* = 1110) were women, the mean age was 46.56 ± 15.26 (20-96) years, 83.26 % attained a junior high school or higher education, 88.77 % were married, and 48.27 % were employed. The rate of smoking was 20.75 %, and drinking was 35.09 %, and 79.75 % of the study subjects reported having regular physical activities. In terms of anthropometric, the mean levels of average BMI, SBP, DBP, WC and FBG for all 1,995 participants were 23.52 ± 3.49 Kg/m^2^, 115.65 ± 17.58 mm Hg, 74.97 ± 10.11 mm Hg, 82.04 ± 10.51 cm, and 5.39 ± 1.32 mmol/L, respectively.

**Table 1:** Socio-demographic and other characteristics of participants in the study

Characteristics	General (*n* = 1995)	Women (*n* = 1110)	Men (*n* = 885)	Statistics	*P*
Age (years)	46.56 ± 15.26	46.07 ± 15.05	47.17 ± 15.53	*t* = 1.594	0.111
BMI (Kg/m^2^)	23.52 ± 3.49	23.21 ± 3.47	23.89 ± 3.46	*t* = 4.320	<0.001
SBP (mm Hg)	115.65 ± 17.58	113.92 ± 18.31	117.81 ± 16.36	*t* = 4.993	<0.001
DBP (mm Hg)	74.97 ± 10.11	73.16 ± 10.06	77.24 ± 9.71	*t* = 9.140	<0.001
WC (cm)	82.04 ± 10.51	79.16 ± 10.14	85.67 ± 9.83	*t* = 14.438	<0.001
FBG (mmol/L)	5.39 ± 1.32	5.28 ± 1.13	5.52 ± 1.52	*t* = 3.937	<0.001
Physical activity, n (%)					
Yes	1591 (79.75)	979 (88.19)	612 (69.15)	χ^2^ = 110.599	<0.001
No	404 (20.25)	131 (11.81)	273 (30.85)		
Smoking status, n (%)					
Ever smoker	414 (20.75)	10 (0.90)	404 (45.65)	χ^2^ = 599.570	<0.001
Never smoker	1581 (79.25)	1100 (99.10)	481 (54.35)		
Drinking habit, n (%)					
Non-drinker	1295 (64.91)	887 (79.91)	408 (46.10)	χ^2^ = 248.308	<0.001
Non-habitual drinker	586 (29.37)	192 (17.30)	394 (44.52)		
Habitual drinker	114 (5.72)	31 (2.79)	83 (9.38)		
Marital status, n (%)					
Single	129 (6.47)	69 (6.22)	60 (6.78)	χ^2^ = 30.648	<0.001
Married	1771 (88.77)	962 (86.67)	809 (91.41)		
Widowed	25 (1.25)	21 (1.89)	4 (0.45)		
Divorced	70 (3.51)	58 (5.22)	12 (1.36)		
Educational level, n (%)					
0 year	73 (3.66)	63 (5.68)	10 (1.13)	χ^2^ = 65.626	<0.001
1–6 years	261 (13.08)	172 (15.50)	89 (10.06)		
7–9 years	524 (26.26)	286 (25.77)	238 (26.89)		
10–12 years	855 (42.86)	475 (42.79)	380 (42.94)		
≥12 years	282 (14.14)	114 (10.26)	168 (18.98)		
Employment status, n (%)					
Unemployed	92 (4.61)	56 (5.05)	36 (4.07)	χ^2^ = 184.955	<0.001
Retired	487 (24.41)	262 (23.60)	225 (25.42)		
Others	156 (7.82)	77 (6.94)	79 (8.93)		
Homemaker	297 (14.89)	269 (24.23)	28 (3.16)		
Employed	963 (48.27)	446 (40.18)	517 (58.42)		

Data are expressed as mean ± SD if not indicated.

*BMI* Body mass index, *FBG* Fasting blood glucose, *WC* Waist circumference

Significant differences were found in different gender groups for BMI, SBP, DBP, WC, FBG, physical activity, smoking status, drinking habit, marital status, educational level, and employment status (Table 1). Subjects were examined for the presence of diabetes and hypertension. Approximately 8.78 % of participants suffered from diabetes and one fifth of subjects (22.31 %) suffering from hypertension (Table 2). Using BMI as the anthropometric indicator, the prevalence of overweight and obesity among the 1,995 participants were 32.03 % and 10.33 %, respectively (Table 2).

**Table 2:** Relationships between related factors and levels of TG, TC, HDL-C and LDL-C in serum samples (mmol/L)

Variables	TC			TG			HDL-C			LDL-C		
	n	Median	Range	n	Median	Range	n	Median	Range	n	Median	Range
Gender												
Men	885	5.09	0.94–14.58	885	1.36^***^	0.34–21.83	885	1.28^***^	0.41–5.24	885	3.26^***^	0.43–7.06
Women	1110	4.95	0.86–13.35	1110	1.12	0.25–10.56	1110	1.47	0.62–4.51	1110	3.07	0.87–7.38
Diabetes												
Yes	175	5.18^*^	1.02–10.49	175	1.56^***^	0.47–21.83	175	1.25^***^	0.68–2.28	175	3.32^**^	1.30–6.50
No	1820	4.99	0.86–14.58	1820	1.20	0.25–21.22	1820	1.39	0.41–5.24	1820	3.14	0.43–7.38
Hypertension												
Yes	445	5.34^***^	1.49–13.35	445	1.57^***^	0.47–11.47	445	1.32^***^	0.60–2.41	445	3.44^***^	1.06–7.38
No	1550	4.93	0.86–14.58	1550	1.14	0.25–21.83	1550	1.40	0.41–5.24	1550	3.07	0.43–7.06
Body size												
Underweight	123	4.51^***^	0.87–9.44	123	0.93^***^	0.25–3.77	123	1.56^***^	1.01–2.72	123	2.63^***^	1.26–6.89
Normal weight	1027	4.89	0.86–14.58	1027	1.05	0.27–21.83	1027	1.46	0.41–5.24	1027	3.03	0.43–7.38
Overweight	639	5.21	0.94–9.99	639	1.52	0.41–20.38	639	1.30	0.63–2.29	639	3.36	1.06–6.99
Obese	206	5.31	1.49–9.14	206	1.80	0.53–13.99	206	1.23	0.60–2.06	206	3.44	1.59–6.43
Age groups (years)												
20–29	269	4.51^***^	0.86–9.57	269	0.96^***^	0.33–13.99	269	1.43^**^	0.74–2.60	269	2.68^***^	1.26–6.80
30–39	545	4.80	0.87–14.58	545	1.10	0.25–21.22	545	1.37	0.74–5.24	545	2.99	0.43–7.06
40–49	434	5.07	1.02–9.44	434	1.27	0.27–20.38	434	1.34	0.41–2.66	434	3.17	1.25–8.89
50–59	316	5.42	0.94–9.31	316	1.40	0.40–11.21	316	1.41	0.63–2.71	316	3.45	1.30–6.99
60–69	251	5.40	5.40–10.49	251	1.46	0.53–21.83	251	1.37	0.62–4.51	251	3.50	1.56–6.50
70–79	150	5.28	1.36–13.35	150	1.34	0.47–4.71	150	1.35	0.83–2.31	150	3.42	2.00–7.38
≥80	30	5.46	2.77–7.94	m	1.20	0.48–7.36	30	1.37	1.00–2.12	30	3.33	1.06–6.18

*: *P* < 0.05; **: *P* < 0.01; ***: *P* < 0.001

*TC* Total cholesterol, *TG* Triglycerides, *HDL-C* High density lipoprotein cholesterol, *LDL-C* Low density lipoprotein cholesterol

### Serum lipid concentrations

The mean concentrations of TC, TG, HDL-C, and LDL-C were 5.11 ± 1.15 mmol/L, 1.59 ± 1.47 mmol/L, 1.42 ± 0.33 mmol/L, and 3.22 ± 0.84 mmol/L, respectively, in the general Shenzhen population aged from 20 to 96 years.

There were significant differences in TG, HDL-C, and LDL-C concentrations between men and women, whereas the mean concentration of TC was similar in both genders (*P* = 0.083). In addition, we found that serum lipid concentrations were significantly positively (including: TC, TG, LDL-C) or negatively (HDL-C) correlated to diabetes, hypertension, body size, and age (Table 2).

### Prevalence of dyslipidemia components

In this study, 31.88 % of the study population showed at least one abnormal lipid parameter. When analyzing for dyslipidemia components, a hypercholesterolemia was found in 14.49 %, a hypertriglyceridemia in 16.14 %, an elevated levels of low density lipoprotein cholesterol in 12.13 %, and abnormally low high density lipoprotein cholesterol in 8.82 % of the study participants (Table 3). Almost one sixths of the study participants (15.99 %) showed more than one abnormal lipid fractions, and in 4 subjects deranged levels of all four lipid fractions was found (data not shown).

**Table 3:** Proportion (95 % confidence interval) of concentrations classification of TC, TG, HDL-C and LDL-C in Shenzhen adults

	Prevalence, % (95 % CI)		
	Overall	Men	Women
Serum TC (mmol/L)			
<5.18	56.19 (54.0–58.4)	53.22 (49.9–56.5)	58.56 (55.6–61.4)
5.18–6.21	29.32 (27.4–31.3)	33.00 (29.9–36.1)	26.39 (23.9–29.0)
≥6.22	14.49 (13.0–16.1)	13.78 (11.6–16.2)	15.05 (13.0–17.2)
Serum TG (mmol/L)			
<1.70	70.33 (68.3–72.3)	63.28 (60.1–66.4)	75.95 (73.4–78.4)
1.70–2.25	13.53 (12.1–15.1)	14.91 (12.7–17.4)	12.43 (10.6–14.5)
≥2.26	16.14 (14.6–17.8)	21.81 (19.2–24.6)	11.62 (9.8–13.6)
Serum HDL-C (mmol/L)			
<1.04	8.82 (7.6–10.1)	14.24 (12.0–16.6)	4.50 (3.4–5.8)
1.04–1.54	62.11 (60.0–64.2)	68.47 (65.4–71.5)	57.03 (54.1–59.9)
≥1.55	29.07 (27.1–31.1)	17.29 (14.9–19.9)	38.47 (35.6–41.4)
Serum LDL-C (mmol/L)			
<3.37	59.80 (57.6–61.9)	55.25 (52.0–58.5)	63.42 (60.6–66.2)
3.37–4.13	28.07 (26.1–30.1)	32.66 (29.6–35.8)	24.42 (21.9–27.0)
≥4.14	12.13 (10.7–13.6)	12.09 (10.1–14.3)	12.16 (10.3–14.2)

*TC* Total cholesterol, *TG* Triglycerides, *HDL-C* High density lipoprotein cholesterol, *LDL-C* Low density lipoprotein cholesterol

The prevalence of dyslipidemia components according to gender, age, body size, and other characteristics is provided in Table 4. Higher hypertrigylceridemia prevalence was found among those men who were ever smoker, central obesity, overweight and obese, and older. The prevalence of high TC, high LDL-C, and low HDL-C was significantly high in those with abnormal body weight. The prevalence of high TC was significantly high among the respondents with alcohol drinking and the aged. The prevalence of low HDL-C was found to be significantly high in men, ever smoker, and those with central obesity, whereas the prevalence of high LDL-C was significantly associated with age.

**Table 4:** The prevalence of dyslipidemia components

Characteristics	N	High TC	High TG	Low HDL-C	High LDL-C
		n (%)	n (%)	n (%)	n (%)
Gender					
Men	885	122 (13.78)	193 (21.81) ^***^	126 (14.24) ^***^	107 (12.09)
Women	1110	167 (15.05)	129 (11.62)	50 (4.50)	135 (12.16)
Smoking status					
Ever smoker	414	65 (15.70)	109 (26.33) ^***^	66 (15.94) ^***^	52 (12.56)
Never smoker	1581	224 (14.17)	213 (13.47)	110 (6.95)	190 (12.02)
Central obesity					
Yes	279	45 (16.13)	85 (30.47) ^***^	44 (15.77) ^***^	41 (14.69)
No	1716	244 (14.22)	237 (13.81)	132 (7.69)	201 (11.71)
Drinking habit					
Non-drinker	1295	118 (9.11) ^**^	203 (15.68)	109 (8.42)	165 (12.74)
Non-habitual drinker	586	83 (14.16)	93 (15.87)	60 (10.24)	65 (11.09)
Habitual drinker	114	18 (15.80)	26 (22.81)	7 (7.02)	12 (10.53)
Body size					
Underweight	123	17 (13.82) ^**^	2 (1.63) ^***^	1 (0.81) ^***^	13 (10.57) ^***^
Normal weight	1027	118 (11.49)	93 (9.06)	50 (4.87)	87 (8.47)
Overweight	639	118 (18.47)	158 (23.62)	84 (13.15)	111 (17.37)
Obese	206	36 (17.48)	69 (30.53)	41 (19.90)	31 (15.05)
Age groups (years)					
20–29	269	17 (6.32) ^***^	18 (6.69) ^***^	17 (6.32)	15 (5.58) ^***^
30–39	545	48 (8.81)	87 (15.96)	55 (10.09)	35 (6.42)
40–49	434	51 (11.75)	75 (17.28)	38 (8.76)	43 (9.91)
50–59	316	73 (23.10)	61 (19.30)	32 (10.12)	59 (18.67)
60–69	251	57 (22.71)	55 (21.91)	23 (9.16)	52 (20.63)
70–79	150	35 (23.33)	23 (15.33)	10 (6.67)	31 (20.67)
≥80	30	8 (26.67)	3 (10.00)	1 (3.33)	7 (23.33)

*: *P* < 0.05; **: *P* < 0.01; ***: *P* < 0.001

*TC* Total cholesterol, *TG* Triglycerides, *HDL-C* High density lipoprotein cholesterol, *LDL-C* Low density lipoprotein cholesterol

### Prevalence and influencing factors of dyslipidemia

Dyslipidemia was found for 636 (31.88 %) of the participants. A positive history of dyslipidemia disease in the past year, and/or currently receiving treatment with lipid-lowering medications was described by 55 subjects, so that the total prevalence of dyslipidemia was 34.64 %. Adjusted for age on the basis of Shenzhen census 2010, the prevalence of dyslipidemia was 25.98 %. This represents 2.2 million Shenzhen adults aged from 20 to 96 years with dyslipidemia. Among subjects with dyslipidemia, 173(25.04 %) were aware of the diagnosis of their condition.

A number of potential independent factors that might influence the prevalence of dyslipidemia were evaluated by univariate logistic regression model. The results revealed that the variables of gender, diabetes, hypertension, central obesity, smoking status, body size, marital status, educational level, employment status, household per capita income (data not shown), and age were significantly associated with the prevalence of dyslipidemia (Table 5). On the contrary, the prevalence of dyslipidemia was found not to be significantly higher in non-habitual drinkers and habitual drinkers, and exercisers. In order to further evaluate the main factors contributed to the prevalence of dyslipidemia and control potential confounding factors, multiple stepwise regression analyses were also performed. Stepwise multiple regression analysis revealed that the presence of dyslipidemia was still significantly associated with increasing age, diabetes, hypertension, body size, and smoking status (Table 6). With regard to health status, participants with diabetes had a 1.498 fold increased prevalence of dyslipidemia (*P* = 0.022), and participants with hypertension had 1.595 fold increased prevalence of dyslipidemia (*P* <0.001). With regard to health behaviors, ever smokers more likely to had dyslipidemia (OR = 2.033, *P* < 0.001). Evaluation of body size suggested that, participants with high body size had a greater prevalence of dyslipidemia (overweight: OR = 3.433, *P* < 0.001; obese: OR = 4.405, *P* < 0.001). Compared with participants 20 to 29 years of age, those who were 50 to 59 years of age had a greater prevalence of dyslipidemia (OR = 3.012, *P* < 0.001).

**Table 5:** Univariate logistic regression analysis of socio-demographic and lifestyle factors associated with dyslipidemia

Characteristics	OR (95 % CI)	*P* value
Gender	1.719 (1.427–2.070)	<0.001
Diabetes	2.502 (1.829–3.423)	<0.001
Hypertension	2.818 (2.271–3.498)	<0.001
Central obesity	2.280 (1.765–2.944)	<0.001
Physical activity	1.057 (0.841–1.328)	0.634
Smoking status	1.923 (1.543–2.397)	<0.001
Drinking habit		
Non-drinker	1 (reference)	
Non-habitual drinker	1.013 (0.825–1.244)	0.901
Habitual drinker	1.025 (0.686–1.532)	0.902
Body size		
Underweight	1 (reference)	
Normal weight	1.613 (0.979–2.660)	0.061
Overweight	4.914 (2.970–8.129)	<0.001
Obese	6.382 (3.674–11.084)	<0.001
Marital status		
Single	1 (reference)	
Married	2.139 (1.376–3.325)	0.001
Divorced	2.641 (1.065–6.551)	0.036
Widowed	3.741 (1.980–7.069)	<0.001
Educational level		
≥12 years	1 (reference)	
10–12 years	0.974 (0.728–1.304)	0.860
7–9 years	1.417 (1.042–1.928)	0.026
1–6 years	1.557 (1.094–2.217)	0.014
0 year	2.064 (1.222–3.487)	0.007
Employment status		
Employed	1 (reference)	
Homemaker	1.263 (0.958–1.666)	0.098
Others	1.390 (0.976–1.981)	0.068
Retired	2.074 (1.655–2.599)	<0.001
Unemployed	0.901 (0.558–1.456)	0.671
Age groups (years)		
20–29	1 (reference)	<0.001
30–39	1.944 (1.346–2.808)	<0.001
40–49	2.333 (1.602–3.396)	<0.001
50–59	4.325 (2.938–6.367)	<0.001
60–69	4.301 (2.875–6.434)	<0.001
70–79	4.020 (2.559–6.316)	<0.001
≥80	3.707 (1.685–8.159)	0.001

**Table 6:** Multivariate stepwise logistic regression analysis of factors associated with dyslipidemia

Characteristics	OR (95 % CI)	*P* value
Diabetes		
No	1 (reference)	
Yes	1.498 (1.061–2.114)	0.022
Hypertension		
No	1 (reference)	
Yes	1.595 (1.234–2.060)	<0.001
Smoking status		
Ever smoker	1 (reference)	
Never smoker	2.033 (1.604–2.577)	<0.001
Body size		
Underweight	1 (reference)	
Normal weight	1.280 (0.763–2.147)	0.351
Overweight	3.433 (2.033–5.798)	<0.001
Obese	4.045 (2.267–7.215)	<0.001
Age groups (years)		
20–29	1 (reference)	
30–39	1.747 (1.189–2.567)	0.004
40–49	1.761 (1.184–2.618)	0.005
50–59	3.012 (1.989–4.563)	<0.001
60–69	2.880 (1.860–4.460)	<0.001
70–79	2.722 (1.657–4.471)	<0.001
≥80	2.280 (0.974–5.339)	0.058

## Discussions

With economic growth and adverse changes in lifestyle (such as a high intake of dietary saturated fat, physical inactivity and smoking), cardiovascular diseases have become the leading cause of death in China [20]. Dyslipidemia is one of the most important independent modifiable risk factors for cardiovascular diseases. A prevention measure against the epidemic of dyslipidemia will undoubtedly depress the morbidity and mortality of cardiovascular diseases.

This study was conducted in 8 districts of Shenzhen to comprehensive assess the prevalence of dyslipidemia as well as the associated risk factors. Our results indicated that the prevalence of high TC, high TG, high LDL-C, and low HDL-C were 14.49 %, 16.14 %, 12.13 %, and 8,82 %, respectively. It was also revealed that the prevalence of dyslipidemia was about 34.64 % (standardized rate per 2010 Shenzhen population: 25.98 %). Furthermore, our results suggested that the presence of dyslipidemia was significantly associated with age, diabetes, hypertension, body size, and smoking status.

Several previous epidemiological studies reported serum lipid concentrations in Chinese populations [9, 21, 22]. In those studies, the China National Diabetes and Metabolic Disorders Study measured serum lipids in a nationally representative sample of 47,352 Chinese aged 20 years or above and provided the best comparison data for our study [22]. When compared with the findings from the China National Diabetes and Metabolic Disorders Study, the mean levels of TC (5.11 mmol/L vs 4.72 mmol/L), TG (1.59 mmol/L vs 1.57 mmol/L), HDL-C (1.42 mmol/L vs 1.30 mmol/L), and LDL-C (3.22 mmol/L vs 2.68 mmol/L) in this study did subtle changes [22]. Also other regional studies have previously examined serum lipid levels in local residents. Wang and co-researchers reported that the mean levels of TC, TG, HDL-C, and LDL-C in Beijing were 5.05 mmol/L, 1.53 mmol/L, 1.50 mmol/L, and 3.27 mmol/L, respectively [23]. Wu et al. reported that the mean levels of TC, TG, and HDL-C in Shanghai were 4.70 mmol/L, 1.50 mmol/L, and 1.40 mmol/L, respectively [24]. Our results were similar to those of the above mentioned studies.

During the past several decades, favorable trends in lipid levels have occurred among adults in the United States [25]. For example, the mean levels of TC declined from 206 to 196 mg/dL between 1988 to 1994 and 2007 to 2010. Additionally, the mean concentrations of LDL-C declined from 129 to 116 mg/dL during 1988-1994 and 2007–2010. On the contrary, compared to the result from the 2002 China National Nutrition and Health Survey, unfavorable trends in lipid levels have occurred among adults in Shenzhen [26]. It is in consistent with the general increase in the mean levels of serum lipid (including: TC, TG, LDL-C) in China with the increasing urbanization and changes in lifestyle [22]. These findings showed important public health implications, which might explain the recent rapid increase in cardiovascular disease burdens in Shenzhen [13].

The prevalence of high TC (14.49 %) and high LDL-C (12.13 %) found in this study were significantly higher than average Chinese national standards (9.0 % and 6.5 %, respectively), whereas the prevalence of low HDL-C (8.82 %) was significantly lower than the national average (22.30 %) [22]. High TC is one of the common lipid disorders in western countries. With the economic growth and the increasing popularity of western foods in Shenzhen, an increasing number of people have changed their dietary patterns. For example, they may choose to consume a more westernized diet, which is high in fat and cholesterol. Therefore, it may be the main contributor to the high TC in Shenzhen. Compared with studies carried out in some other regions, the prevalence of dyslipidemia components in Shenzhen were lower than those in Shanghai [24], Delhi [14], and Turkish [1], but a higher prevalence of high TC or high TG, and lower prevalence of high LDL-C or low HDL-C were found in Shenzhen than in Shaoxing [12]. The reported prevalence of dyslipidemia components varied among Shenzhen, Shanghai, Delhi, Turkish and Shaoxing, depending on the information available for the given population and the diagnostic criteria used. In the present study, the most prevalent component of dyslipidemia was the increased TG with a prevalence of 16.14 %. A similar study reported that the most prominent change in serum lipids in Beijing and Guangzhou over the past 20 years was the increase in serum TG levels, which were dramatically influenced by the increased consumption of diets high in simple carbohydrates and fat [27]. However, other studies found that the major types of dyslipidemia in China and other Asian countries are low HDL-C [24, 28, 29], whereas high TC as the most common lipid disorder in Western countries [1].

The prevalence of dyslipidemia in our study was partially comparable to figures reported in previous epidemiological studies [10, 12, 24]. For example, in a cross sectional study with a representative sample of 5,761 Chinese adults aged from 18 to 79 years, 35.40 % of the subjects had dyslipidemia [10]. In addition, in a recent study on 11,877 Shaoxing residents aged 20 years or older, the age standardized prevalence of dyslipidemia was 25.80 % (the age standardized dyslipidemia prevalence in Shenzhen was 25.98 %) [12]. Our results, together with other study, confirmed that the prevalence of dyslipidemia is increasing in Shenzhen [22, 26]. Notably, the rate of awareness documented in this survey is lower than those recently reported from cross sectional study conducted in Beijing, and elsewhere during the same periods [30, 31]. It is obviously that Shenzhen is going to experience a large increase in the prevalence of cardiovascular disease, if reasonable and effective control measures not taken.

Previous studies reported that age did appear to influence dyslipidemia risk [1, 11, 12, 14, 24]. In the present study, compared with participants aged from 20 to 29 years, those who were 50 to 59 years of age had a greater prevalence of dyslipidemia. With the aging population trend in China, the picture of the public health posed by dyslipidemia will be aggravated. However, the precise mechanisms of the effect of age on serum lipid concentrations are not exactly known—they may be related to hereditary characteristics and degenerative processes as well as creeping weight gain and progressive development of insulin resistance [1]. In addition, the prevalence of dyslipidemia increased with BMI in our study, which was consistent with some previous studies [1, 14, 32, 33]. The lipid abnormalities in overweight and obesity is likely a consequence of insulin resistance [34]. The overweight and obesity prevalence in 2010 was 30.6 % and 12.0 %, which have increased by 34.21 % and 69.01 %, respectively, since 2002 [35, 36]. By comparison, we found that the prevalence of abnormal body weight in Shenzhen was comparable with that in mainland China as a whole [35]. The rapid development of overweight or obesity in Shenzhen will undoubtedly accelerate the prevalence of dyslipidemia.

Several well established risk factors for dyslipidemia, including hypertension and diabetes have been evaluated [1, 12, 14, 33, 37]. We found a consistent pattern of an increasing prevalence of dyslipidemia with increasing fasting blood glucose, and blood pressure. Several factors are likely to be responsible for diabetic dyslipidemia: insulin effects on liver apoprotein production, regulation of lipoprotein lipase, actions of cholesteryl ester transfer protein, and peripheral actions of insulin on adipose and muscle [38]. Previous studies have demonstrated that genetic factors may influence development of dyslipidemia hypertension, nongenetic, potentially modifiable aspects of obesity are also closely related to expression of this clinically important syndrome [39]. Given the relative high prevalence of dyslipidemia in patients with hypertension or diabetes, it is imperative that any patient with hypertension or diabetes be screened for dyslipidemia.

Other researchers observed associations between dyslipidemia and smoking [11, 40, 41]. Our results also showed that smoking status of the participants was related to the prevalence of dyslipidemia. Concentrations of TG in current smokers were shown to be significantly higher and concentrations of HDL-C lower than those who had never smoked or had stopped smoking [41]. Chitra et al. reported the presence of carbon monoxide and nicotine content in the cigarette smoke, which decreases the activity of lipoprotein lipase resulted in the elevated levels of triglycerides [42].

Potential limitations of our study should be mentioned. First, no dietary intake was measured. Therefore, our study cannot show their effect on serum lipids. Second, potential recalled sources of bias including recall bias and self-reported information. Finally, other risk factors (such as stress, genetic factors) were not taken into account.

## Conclusions

Dyslipidemia was present in about 34.64 % of the study population in Shenzhen, and the awareness rate of dyslipidemia was 25.04 %. Risk factors associated with dyslipidemia were aging, higher body size, hypertension, diabetes, and smoking. Dyslipidemia is becoming an important public health problem. Obesity can be used to screen for dyslipidemia along with other coexisting risk factors such as hypertension and diabetes. There is an urgent need to institute more positive public health measures and screening programs and to treat patients diagnosed with dyslipidemia.
